# Technical Verification and Assessment of Independent Validation of Biomarker Models for Endometriosis

**DOI:** 10.1155/2019/3673060

**Published:** 2019-07-25

**Authors:** Dorien F. O, Amelie Fassbender, Rita Van Bree, Annouschka Laenen, Daniëlle P. Peterse, Arne Vanhie, Etienne Waelkens, Thomas M. D'Hooghe

**Affiliations:** ^1^KU Leuven Department of Development and Regeneration, Woman and Child, 3000 Leuven, Belgium; ^2^Department of Obstetrics and Gynecology, Leuven University Fertility Center, University Hospital Leuven, 3000 Leuven, Belgium; ^3^KU Leuven Department of Public Health and Primary Care, Leuven Biostatistics and Statistical Bioinformatics Centre (L-BioStat), 3000 Leuven, Belgium; ^4^SYBIOMA, Facility for Systems Biology Based Mass Spectrometry, 3000 Leuven, Belgium; ^5^KU Leuven Department of Cellular and Molecular Medicine, 3000 Leuven, Belgium

## Abstract

There is a great need for a noninvasive diagnosis for endometriosis. Several biomarkers and biomarker panels have been proposed. Biomarker models consisting of CA-125, VEGF, Annexin V, and glycodelin/sICAM-1 were previously developed by our group. The objective of our current study was to assess the impact of technical and biological variability on the performance of those previously developed prediction models in a technical verification and a validation setting. The technical verification cohort consisted of peripheral blood plasma samples from a subset of the patients included in the original study of Vodolazkaia* et al.* (99 women with and 37 women without endometriosis). The validation study was done in plasma samples of an independent patient cohort (170 women with and 86 women without endometriosis). Single immunoassays were used for CA-125, VEGF-A, sICAM-1, Annexin V, and glycodelin. Statistical analyses were done using univariate and multivariate (logistic regression) approaches. The previously reported prediction models for endometriosis had a low performance in both the technical verification and validation setting. New prediction models were developed, which included CA-125, Annexin V, and sICAM-1, but CA-125 was the only marker that was retained in the models across the technical verification and validation study. Overall, successful validation of a biomarker model depends on several factors such as patient selection, collection methods, assay selection/handling, stability of the marker, and statistical analysis and interpretation. There is a need for standardized studies in large, well-defined patient cohorts with robust assay methodologies.

## 1. Introduction

Endometriosis is a benign gynaecological disorder defined as the presence of endometrial-like tissue outside the uterus, affecting about 10% of women of reproductive age [1] and up to 35-50% of women with chronic pelvic pain and/or infertility [1, 2]. It is staged by the classification system of the American Society for Reproductive Medicine (Stage I: minimal, II: mild, III: moderate, IV: severe) [3]. Due to the overlap in symptoms with other diseases, endometriosis cannot be diagnosed based on the symptoms of pelvic pain and infertility alone [4]. Vaginal ultrasound is an adequate method to detect endometriotic ovarian cysts but does not rule out peritoneal endometriosis or endometriosis-associated adhesions [5]. The gold standard for diagnosis is laparoscopic visualization of the lesions with histological confirmation [6]. Several studies have reported diagnostic delays in endometriosis averaging between 8 and 11 years [7]. Noninvasive diagnosis of endometriosis would allow early diagnosis and treatment, with the potential to improve quality of life and to reduce the costs related to endometriosis [8]. A test with high sensitivity is needed, with a low number of false negative results,* i.e.*, a low number of patients who have a negative test but who do have endometriosis [9]. Such a test would especially be useful for women with pelvic pain and/or infertility with a normal ultrasound [9].

Several biomarkers and biomarker panels have been proposed for endometriosis [10–13]. Our group has previously reported a high diagnostic value of plasma biomarkers for endometriosis. Two panels of 4 biomarkers (Annexin V, VEGF, CA-125, and glycodelin/sICAM-1) [11], measured in plasma samples obtained during menstruation, allowed the detection of ultrasound (US-) negative endometriosis with high sensitivity (82%) and acceptable specificity (75%) [11]. In the same study, menstrual plasma levels of 3 biomarkers (VEGF, Annexin V, and CA-125) allowed the diagnosis of endometriosis (stages I-IV, both with and without US evidence) with 85% sensitivity and 75% specificity [11]. However, it is important to note that no biomarker or biomarker panel for endometriosis has been validated for clinical application in peripheral blood [13], nor in endometrium [14].

This lack of biomarker validation can be attributed to different types of variation that interfere with the interpretation of biological results, namely, preanalytical, technical, and biological variation [15, 16]. Firstly, preanalytical variation occurs during sample collection, processing, and storage. To overcome this variation and allow large multicentric studies, the World Endometriosis Research Foundation (WERF) has developed the Endometriosis Phenome and Biobanking Harmonization Project (EPHect), which provides standard operating procedures (SOPs) for the handling of fluid and tissue for biobanking purposes [17, 18]. A second important aspect of biomarker research which is often overlooked in the endometriosis field is the investigation of assay robustness and reproducibility across different laboratories [15]. One of the milestones of the biomarker clinical validation process is the validation of initial findings with a clinical assay that replaces the biomarker discovery assay [19]. Technological platforms differ widely in assay variability and diagnostic accuracy. Substantial differences in analyte levels can be found when assay kits from different manufacturers are used or even in different lots of assay kits supplied by single manufacturers [20]. Variability can be induced by the use of different standards, antibodies, and the quality of the lab performance [20] and approaches of statistical analyses [19]. Thirdly, natural biological variation, due to differences in disease severity and phenotype but also due to confounding factors, impacts biomarker performance. In an effort to address this issue, WERF has released questionnaires and a surgical sheet for surgical and clinical phenotyping of patients [21, 22]. To assess the relevance of a biomarker outside of the initially tested sample cohort, it is essential to test it in an independent patient set.

The general objective of our current study was to assess whether technical and biological variation affect the performance of the biomarker models developed by Vodolazkaia* et al.* [11]. To fulfill this objective, we have performed experiments in two settings: (1) a technical verification study = selection of a subset of patients included in the Vodolazkaia sample cohort, followed by analysis of these samples in a different laboratory using partially different immunological assays (Roche Diagnostics, Penzberg, Germany) to assess reproducibility and (2) a validation study = selection of an independent sample cohort including women during the menstrual phase of the cycle, but also additional sample cohorts of women in the follicular/luteal phase of the cycle or using hormonal medication and using four out of five assays originally used in the Vodolazkaia study [11], with analysis performed in our laboratory in Leuven, Belgium.

The aim of the present study was to assess univariate analysis and to reapply the prediction models (independent variables: Annexin V, VEGF, CA-125, and glycodelin/sICAM-1) developed by Vodolazkaia* et al.* [11] on plasma samples from patients in the menstrual phase of the cycle in both settings (technical verification study and validation study). Our second aim was to investigate in both settings how the same previously [11] identified biomarkers (Annexin V, VEGF, CA-125, and glycodelin/sICAM-1) could be used to develop a new model in samples from patients regardless of menstrual cycle phase and from patients using hormonal medication.

## 2. Materials and Methods

### 2.1. Sample Processing and Patient Selection

Since 1999 a biobank has been developed based on collection and storage of plasma samples from women undergoing laparoscopy for infertility and/or pelvic pain at the Leuven University Fertility Center (LUFC, Belgium). For each patient, detailed clinical information is available in the electronic database, including age, menstrual cycle phase at surgery, a detailed surgery report with scoring and staging of endometriosis according to the classification of the ASRM [3], medication use, and data of preoperative ultrasound [11]. All patients had signed a written informed consent and the study protocol was approved by the Medical Ethics Committee UZ KU Leuven / Research (ML11333 and ML10837).

Plasma samples had been collected at the time of surgery before anesthesia according to our standard operation procedures (SOPs) in EDTA tubes, centrifuged at 1400 g for 10 minutes at 4°C, aliquoted, labelled, and stored at -80°C till analysis [9]. The time interval between sample collection and storage in the -80°C freezer was maximum 1 hour as described in the WERF EPHect SOPs for collection, processing, and storage of blood specimens [17].

#### 2.1.1. Technical Verification Study

The electronic biobank database of the LUFC was searched for all patients that had been selected in a previous study by our group conducted by Vodolazkaia and coworkers [11]. Only patients with the minimal required volume of plasma (1 ml) were selected. None of the selected sample aliquots had previously been thawed. The samples had been collected between 2001 and 2010. Of the 353 originally selected patients [11], 136 had plasma available and were used in our present study. Plasma samples from patients using hormonal medication (combined oral contraceptive pill or progestins or GnRH analogues) and from patients operated within 6 months prior to the time of sample collection had been excluded. These 136 available plasma samples (Table 1) were obtained from 99 women with endometriosis and 37 women without endometriosis. A subset analysis was done on samples collected from 81 women with laparoscopically confirmed endometriosis without evidence of endometriosis on a preoperative gynaecological ultrasound (= US-negative endometriosis).

#### 2.1.2. Validation Study

The electronic biobank database of the LUFC was searched for all patients that had not yet been selected in the previous study by our group conducted by Vodolazkaia and coworkers [11]. Only patients with the necessary clinical information and with the minimal required volume (1 ml) of plasma available were selected. None of the selected plasma aliquots had previously been thawed. The samples had been collected between 2001 and 2016. 256 plasma samples were available (Table 1) from 170 women with endometriosis and 86 women without endometriosis. Samples had been collected in different phases of the menstrual cycle and also from women using combined oral contraceptives (COC) or progestogens. A subset analysis was done on samples collected from 116 women with laparoscopically confirmed endometriosis without evidence of endometriosis on a preoperative gynaecological ultrasound (= US-negative endometriosis).

### 2.2. Determination of Biomarker Levels

#### 2.2.1. Technical Verification Study

All samples selected for the technical verification study were transported on dry ice with temperature monitoring to the laboratories of Roche Diagnostics GmbH, Penzberg, Germany, where analyses were carried out. The technicians who performed the analysis were blinded to the patients' diagnoses. Out of the five assays used, two were the same as those used in our previous study [11] and three were different (Table 2).

CA-125 was measured on a* cobas® e* 601 instrument using commercially available assays; both instrument and assays were developed by Roche (Roche Diagnostics GmbH, Penzberg, Germany). VEGF-A was measured on the same instrument using internal research assays. sICAM-1 was measured with an immunoassay using the IMPACT technology [24]. Plasma levels of Glycodelin were determined with an internally developed ELISA assay (Roche Diagnostics GmbH, Penzberg, Germany), and Annexin V was measured using a commercially available ELISA kit (American Diagnostica GmbH (now Sekisui Diagnostics GmbH), Pfungstadt, Germany).

#### 2.2.2. Validation Study

For the validation study, samples were analyzed in-house at KU Leuven (Belgium). We used the same assays as Vodolazkaia* et al*. [11], except for VEGF for which we chose a single ELISA because it had been part of a Bioplex multiplex immunoassay (Biorad Laboratories, Hercules, CA, USA) in the original study. CA-125 was measured on a Roche Modular E170 instrument using commercially available assays (Roche Diagnostics GmbH, Penzberg, Germany). VEGF-A was measured using a single ELISA (Cloud-clone corp, Houston, USA). sICAM-1 was measured with a Quantikine ELISA from R&D Systems (Minneapolis, MN, USA). Plasma levels of Glycodelin were determined with a single ELISA from Bioserv Diagnostics (Rostock, Germany) and Annexin V was measured using a commercially available ELISA kit (American Diagnostica GmbH (now Sekisui Diagnostics GmbH), Pfungstadt, Germany).

### 2.3. Statistical Analysis

#### 2.3.1. Univariate Analysis

Median and interquartile range were used to describe the data. For the univariate analysis, differences between biomarker levels between cycle phases were analyzed using the Kruskal-Wallis test and Mann-Whitney *U* test for pairwise comparisons. Spearman correlation was used to compare the results of the technical verification study and previous study [11]. A Spearman r between 0 and 0.30 was interpreted as negligible correlation, 0.30 and 0.50 as low correlation, 0.50 and 0.70 as moderate correlation, 0.70 and 0.90 as high correlation, and 0.90 and 1.0 as very high correlation. Analyses were performed using Graphpad prism software (GraphPad Software, San Diego, CA, USA).

#### 2.3.2. Evaluation of Existing Diagnostic Models on Technical Verification and Validation Study

The prediction models with coefficients from Vodolazkaia* et al.* [11] were applied to the new datasets of the technical verification and validation studies to calculate a risk prediction score of each individual patient. ROC curve analysis of these risk prediction scores provides a C-index (area under the ROC curve), which is a measure of model performance.

#### 2.3.3. Development of New Diagnostic Models

A stepwise model selection procedure was followed, with 5% significance level for variables entering in or removal from the model. The C-index (area under the ROC curve) is estimated as a measure for model performance. This index indicates the discriminative power of a model and ranges between 0.5 (discrimination no better than chance) and 1 (perfect discrimination). Cut-offs were chosen to maximize sensitivity for acceptable specificity [25], which was set at 60% or more. Complete-case analyses were performed. Patients receiving hormonal medication were excluded from model building. Analyses were performed using SAS software (version 9.4 of the SAS System for Windows).

## 3. Results

### 3.1. Correlation of Measurements from the Technical Verification Study and Previous Study [11]

**Figure 1:**
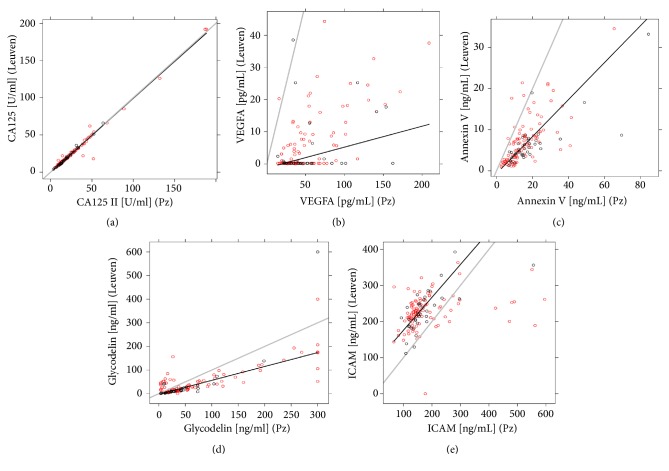
*Immunoassay measurements for (a) CA-125, (b) VEGF, (c) Annexin V, (d) glycodelin and (e) sICAM-1*. Regression lines (black) illustrate the correlation between the measurements from Roche Diagnostics GmbH (Pz, x-axis) and the measurements from Vodolazkaia* et al*. (Leuven, y-axis) for samples from 136 patients. Grey diagonal line represents identity line (100% agreement between assays). Red circles represent endometriosis cases and black circles represent controls.

The technical verification study consisted of a subset of samples (same patient, different aliquot) that had already been measured in a previous study [11], but with other immunological assays in a different laboratory. Therefore, we assessed whether the biomarker measurements correlated between both studies (Table 3 and Figure 1).  Figure 1(a) illustrates the agreement in absolute values of CA-125 between previous and technical verification study measurements. This high level of agreement was further reflected by a Spearman correlation coefficient of 0.97 (Table 3). In contrast, the absolute plasma values of VEGF did not correlate well with the results of the previous study (Figure 1(b); Spearman r = 0.42). Annexin V levels were overall higher in the technical verification study with obvious scatter (Figure 1(c)) but showed a high correlation (r = 0.72). Glycodelin (Figure 1(d)) values were also mostly higher in the technical verification study, except for a group of measurements that was higher in the previous study. Glycodelin only showed moderate correlation between study results (r = 0.63). sICAM-1 values were lower in the technical verification study with obvious scatter when compared to the previous measurements (Figure 1(e)) and had a low-moderate correlation between study results (r = 0.51).

### 3.2. Univariate Analysis of Technical Verification and Validation Study

The data were first analyzed regardless of cycle phase, then according to menstrual cycle phase (menstrual, follicular, and luteal) both for all endometriosis patients and for the subgroup of patients with endometriosis undetectable on a preoperative ultrasound (US-neg).  Table 4 summarizes the results of patients with “all endometriosis” versus the control group. For results of patients with ultrasound-negative endometriosis, see Supplementary Table I.

CA-125 was the only biomarker that showed both in the technical verification study and the validation study a significantly higher value in the endometriosis group, compared with the control group. Glycodelin was also significantly upregulated in the endometriosis group, but only in the validation study. When analyzing according to cycle phase, a significant difference between cases and controls was found for CA-125 in the follicular (technical verification and validation study), luteal (validation study), and medication (validation study) cohort. For Annexin V and sICAM-1 a significant downregulation was found in endometriosis cases taking hormonal medication, when compared with control patients also under hormonal treatment.

### 3.3. Reapplication of the Prediction Models Developed by Vodolazkaia et al.

#### 3.3.1. Based on Original Results Measured by Vodolazkaia et al. [11]

To discern whether the change in cohort composition affected model performance, we applied the prediction models previously developed by Vodolazkaia* et al.* [11] on the original measurements performed by Vodolazkaia* et al.* but only for the subcohort of patients included in the technical verification study. The C-index dropped slightly when compared to the values reported by Vodolazkaia* et al.* but remained significant. For the model diagnosing all menstrual endometriosis (CA-125, VEGF, Annexin V) the C-index was 74.7% (previously reported by Vodolazkaia* et al.* as 69%/80% in train/test set), while for the ultrasound-negative models (CA-125, VEGF, Annexin V, and glycodelin or ICAM), the C-index was 76.0% and 70.6%, respectively (previously reported by Vodolazkaia* et al.* as 81/78% and 79/78%, respectively).

#### 3.3.2. Based on Measurements of the Technical Verification Study

When the prediction models developed by Vodolazkaia* et al.* were applied to the measurements of the technical verification study, these models showed reduced C-indexes. For the model diagnosing all menstrual endometriosis (CA-125, VEGF, Annexin V) the C-index was 63.7%. For the ultrasound-negative models (CA-125, VEGF, Annexin V, and glycodelin or ICAM), the C-index was 64.0% and 53.3%, respectively. Moreover, for none of these models the C-index was significantly larger than 50%, which indicates a nondiscriminatory model.

#### 3.3.3. Based on Measurements of the Validation Study

In the validation cohort the risk prediction score of each patient approached 1, implying that each study participant (both women with and without endometriosis) had an extremely high risk of having endometriosis according to the prediction model. Such a scenario occurs when the model coefficients are not adequate to assess the independent cohort, thereby impeding interpretation of the model.

### 3.4. Development of New Prediction Models

#### 3.4.1. Technical Verification Study

The stepwise selection procedure did not allow construction of a new biomarker model in the menstrual phase of the cycle, likely due to the small sample size. For all phases combined, a model could be built on all patients, but not on US-negative patients, probably due to a lower sample size in the latter group. This model for all patients included both CA-125 and Annexin V and had a C-index (area under the ROC curve) of 68.5% (95% CI: 59.0-78.0%) (see Table 5). At a cut-off of 0.7187, this resulted in a sensitivity of 62.6% and a specificity of 59.5%.

#### 3.4.2. Validation Study

The prediction model containing CA-125 and Annexin V that was developed in the technical verification study was applied to the patients of the validation study (no medication, all cycle phases) but only showed a C-index of 62.3% (95% CI: 54.4-70.2%). To investigate whether this model could be improved by building a new model and whether both markers would be chosen in this new study cohort, we repeated the model building step on the validation cohort.

As in the technical verification study, the stepwise selection procedure did not allow construction of a new biomarker model in the menstrual phase of the cycle. For all phases combined, a model could be built on all patients (excluding patients on hormonal treatment) which included only CA-125, but not Annexin V (Table 5). At a cut-off of 14.0 U/ml this resulted in a sensitivity of 75.6% and a specificity of 63.4%. The area under the ROC curve (C-index) was 73.3% (95% CI: 66.1-80.5%), which was a better performance than the reapplication of the model (CA-125 and Annexin V) developed in the technical verification phase. To assess the impact of hormonal medication on CA-125 model performance, the model was applied on an independent set of patients under oral hormone contraceptives which resulted in a C-index of 75.2% (95% CI: 60.6-89.7%).

In the subgroup of ultrasound-negative patients, a model was built which included CA-125 and sICAM-1 (Table 5). This model had a C-index of 69.8% (95% CI: 61.7-77.8%). At a cut-off of 0.5566 this resulted in a sensitivity of 64.1% and a specificity of 61.4%. When this model was applied to an independent set of patients under oral hormone contraceptives, the C-index was 77.0% (95% CI: 60.7-93.2%).

## 4. Discussion

In this study, we have reapplied the previously developed models from Vodolazkaia* et al.* [11] in two settings: a technical verification study using different immunological platforms in a different laboratory (Penzberg, Germany) and a validation study using an independent patient cohort in the original laboratory (Leuven, Belgium). We did not succeed in validating these previously reported diagnostic models for endometriosis. Our inability to confirm the models in the technical verification study indicate that a change of laboratory environment and assay technology has a fundamental impact, not only on univariate analysis but also on the performance and reproducibility of multivariable biomarker models. This finding however does not rule out the potential usefulness of the previously discovered biomarkers for the diagnosis of endometriosis. Development of new models in the technical verification and validation studies showed that out of the five investigated proteins, only CA-125 was systematically selected by the biomarker selection algorithm using strict selection criteria in “all phase” endometriosis models.

Our study is the first in the endometriosis biomarker field to assess an existing biomarker model on the level of technical variability and patient heterogeneity, which are both known to impact model performance. Our study differentiates itself from other endometriosis biomarker studies by several novel approaches: firstly, the inclusion of a technical verification step where a subset of the same patient samples, used in the original study [11], was reanalyzed with other immunological assays in another laboratory to estimate the reproducibility,* i.e.*, the impact of a change in assay type and laboratory environment on univariate and multivariate analysis. Secondly, we aimed to further validate the original diagnostic models [11] in additional, independent patient cohorts. These steps in the verification/validation pipeline are often neglected in endometriosis research. In fact, the sequence of steps necessary for validation and translation of a promising biomarker to the clinic is unclear in biomarker research, not only in endometriosis but also in the cancer field [26]. Thirdly, an additional strength of our study is the inclusion of patient samples from our large endometriosis biobank which includes full characterization of patients and which operates under strict SOPs for samples collection [9]. Thereby, we can exclude preanalytical variability as an important influence on marker measurements. Fourthly, we included patients under hormonal medication which is a largely underrepresented patient group in endometriosis biomarker research but is an important group of women coming into the clinic presenting with pelvic pain symptoms.

A limitation of our study is the use of different assays between the technical verification and the validation study. For the technical verification study, we had access to assays that were not commercially available, but as part of a collaboration project with Roche Diagnostics GmbH (Penzberg, Germany). Since those Roche assays were not available for the validation study, we had to revert to the assays used by Vodolazkaia* et al.* [11]. Since VEGF had been measured as part of a multiplex immunoassay in the original study [11], we chose to replace this discovery test by a single commercially available ELISA (Cloud-clone corp, Houston, USA) based on previous experience from other research groups in our laboratory. The second limitation of our study is the low sample size, which prevented us from dividing our patient groups according to menstrual cycle phase or disease phenotype as this would affect statistical power. In addition, this low sample size prevented us from splitting our data into a training set and an independent test set, an internal validation method often used for assessment of model performance [26].

Many biomarkers for endometriosis have been investigated [13], but most results remain controversial. Panels of biomarkers have received much attention as they are expected to perform better than single markers for a complex disease such as endometriosis, but multivariable biomarker models are prone to overfitting and the reported models have not been established in independent patient cohorts [27]. The most frequently investigated single protein biomarker in endometriosis has been CA-125, which is a nonspecific tumor marker for a large proportion of epithelial ovarian cancers [28]. This marker is part of the risk of malignancy algorithm (ROMA) and OVA1 diagnostic tests which evaluate the risk of ovarian cancer based on the combination of CA-125 with other biomarkers [29]. As in cancer, consensus exists that CA-125 lacks both sensitivity and specificity for endometriosis [30] and is therefore useless as a standalone diagnostic test for endometriosis [7, 27]. CA-125 was included in the meta-analysis by Nisenblat* et al.* where it was investigated at several cut-off levels [27]. Studies that employed a CA-125 cut-off >10-14.7 U/ml had a mean sensitivity of 70% and a mean specificity of 64% [27]. This corresponded well with our validation study where the chosen cut-off (maximal sensitivity for a > 60% specificity) of 14 U/ml yielded a sensitivity of 75.6% and a specificity of 63.4%. In our study, CA-125 was the most robust marker and the only marker that was selected in both the technical verification and validation study models with reasonable sensitivity and specificity, albeit too low for a replacement or triage test for endometriosis [27]. Therefore, more research should be invested in evaluating the diagnostic accuracy of biomarker panels including CA-125 with other markers.

The reasons for our failure to validate the previously discovered models can be attributed to the effect of two variables on model performance: firstly on a technical level regarding the use of different immunoassays and secondly on a patient level with regard to baseline phenotype heterogeneity and sample size.

Firstly, the level of technical variability could be assessed in our technical verification study where we selected a subgroup of biobanked plasma samples from our previous study [11] on the basis of their availability. By reanalyzing the samples in a different laboratory and using partially different technological assays, we could directly evaluate the impact of differences in assay platforms and handling during sample analysis, while preanalytical sample conditions related to collection methods remained unchanged. Furthermore, by applying a predefined biomarker model, developed in our previous paper [11], we could assess the performance of the statistical models after these changes. Our technical verification study showed that different assays greatly influence the quantification of most biomarkers, in particular VEGF, which leads to loss of model performance. Indeed, only when measurements from two assays are highly correlated, the values of the new assay can be substituted into a model built using measurements from an earlier assay [31]. Interestingly, this high correlation was only found for CA-125 values which were extremely stable across the three studies (Vodolazkaia, technical verification, and validation study), indicating the robustness of this immunological assay and stable levels of the marker. The reproducibility of the assay may be one of the reasons why this protein was selected for model building in the three studies. In contrast, for proteins measured with different or unstable assays, artefacts or technical variability in biomarker measurements may obscure real biological results [15]. This observation emphasizes the need to carefully address the development from a discovery assay to a robust diagnostic assay, an area that has been largely ignored in endometriosis biomarker research.

Secondly, patient selection is very important when assessing diagnostic studies. In endometriosis, patient heterogeneity may arise from selection of patients in different phases of the cycle, stages of endometriosis, disease phenotypes, and confounding factors. In addition, the choice of an adequate control group is crucial. This possibility to divide patients in a large set of subgroups can lead to very small sample sizes in the smallest group. This in turn can have an impact on multivariate analysis of biomarkers as it leads to model overfitting, which is an underestimated cause of failure of diagnostic models. As a rule of thumb, 10 patients should be included per biomarker in each smallest patient group [32, 33].

In the future, biomarker studies for endometriosis should be set up with attention to patient selection, assay design/reproducibility, and statistical methods. Biomarker discovery and validation studies require large and well-characterized patient cohorts. The issue of assay variability could be solved by using standard platforms with low variation in which a large patient cohort is to be investigated in both a training and an independent test set. For biomarker models, it is important to avoid model overfitting and to encourage publication of the model coefficients so that other groups can try to replicate the data.

In conclusion, our study assessed existing biomarker models [11] on the level of technical variability and patient heterogeneity that are both known to impact model performance. This was done in a technical verification and validation approach that is unique in endometriosis research. We did not succeed in validating our previously reported diagnostic models for endometriosis [11]. This finding however does not rule out the potential usefulness of the previously discovered biomarkers for the diagnosis of endometriosis. Overall, successful validation of a biomarker model depends on several factors such as patient selection, collection methods, assay selection/handling, stability of the marker, and statistical analysis and interpretation. There is a need for standardized studies in large, well-defined patient cohorts with robust assay methodologies. It seems likely that in any biomarker panel for endometriosis, CA-125 would be included. This could be in association with other protein markers (such as Annexin V or VEGF), or possibly with biomarkers newly discovered by proteomics, transcriptomics, or miRNAomics.

## Figures and Tables

**Table 1:** Clinical characteristics of selected patients in the technical verification and validation study.

	*Technical verification study* ^1^			*Validation study*		
	Control	Endometriosis	P-value^2^	Control	Endometriosis	P-value^2^
*Numbers*	*n* = 37	*n *= 99		*n* = 86	*n* = 170	
		(US-negative *n* = 81)			(US-negative *n* = 116)	

*Age* (years)						
Mean ± SD	30.8 ± 5.2	31.7 ± 4.0	0.4063	30.3 ± 5.3	31.0 ± 4.6	0.1411
Median, range	31, 19-44	31, 24-42		30, 18-42	31, 14-42	

*Symptoms* (n)						
Subfertility	35	93 (76)	0.8851	70	148 (102)	0.2287
Dysmenorrhea	24	66 (54)	0.8433	53	137 (95)	*0.0011*
Dyspareunia	7	30 (22)	0.1843	26	48 (31)	0.7392
Chronic pelvic pain	2	7 (6)	0.7281	12	37 (31)	0.1335
Dyschezia	3	11 (6)	0.6080	11	17 (10)	0.4992

*Cycle phase* (*n*)						
Menstrual	10	19 (15)	0.3208	17	31 (20)	0.7667
Follicular	13	42 (37)	0.4408	29	42 (30)	0.1281
Luteal	14	38 (29)	0.9535	25	55 (44)	0.5925
Medication	/	/	N/A	15	42 (22)	0.1870

*Stage* (*n*)						
Stages I-II	N/A	71 (71)	N/A	N/A	101 (92)	N/A
Stages III-IV		28 (10)			69 (24)	

*Other pelvic patholog*ies (n)						
Non-endometriotic adhesions	12	/	N/A	26	/	N/A
Myoma	5	6 (6)	0.1560	7	9 (5)	0.3744
Parasalpingeal cyst	10	14 (14)	0.0794	2	4 (4)	0.9891
Hydrosalpinx	4	2 (2)	*0.0263*	5	1 (1)	*0.0090*

N/A = not applicable

^1^ The patients in the technical verification study are a subset of the patient cohort that had been selected by Vodolazkaia *et al.*

^2^ A Mann-Whitney *U* test was used for comparison of endometriosis cases versus controls for continuous variables (age) and chi-square test for categorical variables

**Table 2:** Overview of immunological assays used in study by Vodolazkaia *et al.* and in the current technical verification and validation studies.

Protein	Assay in original study [11]	Assay in technical verification study (collaboration with Roche Diagnostics)	Assay in validation study (in-house at KU Leuven)	Use of the same assay between studies
CA-125	Roche Modular E170 Roche Diagnostics GmbH	cobas e 601 Roche Diagnostics GmbH	Roche Modular E170 Roche Diagnostics GmbH	Yes (successor system)

VEGF-A	Bioplex multiplex immunoassay, BioRad Laboratories, Hercules, USA	cobas e 601 Roche Diagnostics GmbH (Internal Research Assay)	ELISA Cloud-clone corp, Houston, USA	No

Annexin-V	ELISA American Diagnostica GmbH Pfungstadt, Germany (now Sekisui Diagnostics GmbH)	ELISA American Diagnostica GmbH Pfungstadt, Germany (now Sekisui Diagnostics GmbH)	ELISA American Diagnostica GmbH Pfungstadt, Germany (now Sekisui Diagnostics GmbH)	Yes

Glycodelin	ELISA Bioserv Diagnostics, Rostock, Germany	ELISA Roche Diagnostics GmbH (Internal Research Assay)	ELISA Bioserv Diagnostics, Rostock, Germany	Only between Vodolazkaia *et al.* and validation study

sICAM-1	ELISA R&D systems, Minneapolis, USA	IMPACT Roche Diagnostics GmbH (Internal Research Assay)	ELISA R&D systems, Minneapolis, USA	Only between Vodolazkaia *et al.* and validation study

**Table 3:** Correlation analysis of biomarker measurements of the technical verification study (*n* = 136) versus the study performed by Vodolazkaia *et al.* [11].

Biomarkers	Spearman r	95% CI
CA-125	0.97	0.96-0.98

VEGF	0.42	0.27-0.55

Annexin V	0.72	0.63-0.80

Glycodelin	0.63	0.51-0.73

sICAM-1	0.51	0.37-0.63

r between 0 and 0.30 is interpreted as negligible correlation, 0.30 and 0.50 as low correlation, 0.50 and 0.70 as moderate correlation, 0.70 and 0.90 as high correlation, and 0.90 and 1.0 as very high correlation [23]

**Table 4:** Levels of plasma biomarkers for endometriosis (all stages) versus controls.

Biomarker	Phase of cycle	Technical verification study			Validation study		
		Control	Endometriosis	p-value	Control	Endometriosis	p-value
CA-125 (U/ml)	All (no med)	15.61	19.28	*∗*	13.00	20.00	*∗∗∗∗*
		(11.38-22.52)	(13.11-31.76)		(9.000- 16.00)	(14.00- 29.75)	

	Menstrual	19.49	20.56	NS	16.00	25.00	NS
		(12.39-27.42)	(14.83-37.39)		(13.00- 23.50)	(13.00- 50.00)	

	Follicular	11.97	18.81	*∗*	11.00	20.00	*∗∗*
		(9.859-16.13)	(12.16-29.44)		(8.000-17.00)	(11.75- 27.50)	

	Luteal	16.99	19.09	NS	12.00	19.00	*∗∗∗*
		(12.62-25.34)	(13.07-30.30)		(9.500- 13.00)	(15.00- 25.00)	

	Medication	N/A	N/A	N/A	9.000	15.00	*∗*
					(5.000- 12.00)	(9.750- 24.25)	

VEGF (pg/ml)	All (no med)	41.44	40.82	NS	404.6	442.9	NS
		(27.49- 58.59)	(28.52- 67.48)		(296.5-545.1)	(299.5-525.8)	

	Menstrual	43.69	44.57	NS	414.3	478.8	NS
		(29.50- 54.13)	(30.62- 68.32)		(364.5-507.0)	(366.7-548.9)	

	Follicular	34.90	40.16	NS	400.0	424.0	NS
		(25.17- 47.24)	(26.24- 61.08)		(278.5-581.5)	(254.4-521.2)	

	Luteal	56.22	38.05	NS	399.3	425.4	NS
		(22.36-98.14)	(27.12-76.66)		(312.3-568.1)	(322.3-512.2)	

	Medication	N/A	N/A	N/A	368.7	376.5	NS
					(217.2-490.4)	(263.4-449.7)	

Annexin V	All (no med)	15.51	12.62	NS	7.322	10.16	NS
(ng/ml)		(11.41-20.56)	(8.860-19.59)		(3.568-49.06)	(3.051-43.65)	

	Menstrual	15.69	16.05	NS	4.519	6.031	NS
		(13.94-18.17)	(9.640-22.31)		(2.031-25.69)	(2.387-26.19)	

	Follicular	15.06	12.67	NS	9.320	11.39	NS
		(11.16-18.06)	(6.565-19.73)		(3.097-24.11)	(3.280-45.55)	

	Luteal	17.13	11.95	NS	17.05	10.40	NS
		(8.490-24.31)	(8.610-18.25)		(3.881-63.47)	(3.364-60.85)	

	Medication	N/A	N/A	N/A	17.41	4.977	*∗*
					(4.489-57.69)	(2.523-11.56)	

sICAM-1 (ng/ml)	All (no med)	154.8	147.7	NS	209.5	201.0	NS
		(137.4-182.1)	(130.9-195.5)		(183.3- 236.2)	(171.0-226.2)	

	Menstrual	176.0	172.4	NS	195.7	201.0	NS
		(135.0-246.9)	(130.9-298.0)		(186.0-230.2)	(167.8-226.7)	

	Follicular	149.0	153.2	NS	207.9	210.9	NS
		(130.7-170.8)	(129.4-204.2)		(171.0-228.9)	(168.3-237.4)	

	Luteal	155.7	144.3	NS	227.4	186.8	NS
		(134.1-177.1)	(130.7-164.6)		(189.1-241.2)	(171.8-216.3)	

	Medication	N/A	N/A	N/A	227.4	182.3	*∗∗*
					(197.7-260.3)	(163.8-204.7)	

Glycodelin	All (no med)	29.06	34.76	NS	3.237	5.612	*∗*
(ng/ml)		(11.90-47.50)	(16.20-85.41)		(1.254-11.30)	(2.250-17.83)	

	Menstrual	45.68	109.7	NS	7.242	19.68	NS
		(37.62-109.1)	(39.79-190.7)		(2.312-19.86)	(10.46-40.30)	

	Follicular	12.87	17.42	NS	1.573	5.191	NS
		(9.850-23.80)	(10.19-34.30)		(0.9660-7.578)	(2.126-9.351)	

	Luteal	31.13	43.26	NS	5.026	3.669	NS
		(11.91-54.83)	(20.29-99.28)		(1.513-11.30)	(1.772-8.920)	

	Medication	N/A	N/A	N/A	0.9502	2.535	NS
					(0.6382-3.436)	(1.032-4.221)	

Data are presented as the median and interquartile range. Mann-Whitney test was performed for all phases combined, while Kruskal-Wallis with post-hoc Dunn's analysis was done when biomarkers were analyzed according to cycle phase. No med = no medication. NS = not significant. N/A indicates that there was no medication cohort included in the technical verification study

**Table 5:** Coefficients and model performance of diagnostic models for endometriosis that were newly developed in patients who did not use hormonal medication in the technical verification and the validation study.

Variable	Model coefficient	P-value	Model	C-index	N patients
			P-value	(95% CI)	
*Technical verification study all phases combined all endometriosis*					

Intercept	0.8288	0.0581	0.0045	0.685 (0.590;0.780)	136
CA-125	0.0378	0.0326			
Annexin V	-0.0387	0.0261			

*Validation study all phases combined all endometriosis*					

Intercept	-0.1320	0.6332	0.0002	0.733 (0.661;0.805)	198
CA-125	0.0342	0.0070			

*Validation study all phases combined US-neg endometriosis*					

Intercept	1.4793	0.0797	0.0023	0.698 (0.617;0.778)	162
CA-125	0.0277	0.0328			
sICAM-1	-0.0086	0.0314			

## Data Availability

The ELISA data used to support the findings of this study are available from the corresponding author upon request.

## Supplementary Materials

Supplementary Materials Supplementary Table I. Levels of plasma biomarkers for ultrasound-negative endometriosis versus controls.

## References

[1] Giudice L. C., Kao L. C. (2004). Endometriosis. *The Lancet*.

[2] Meuleman C., Vandenabeele B., Fieuws S., Spiessens C., Timmerman D., D'Hooghe T. (2009). High prevalence of endometriosis in infertile women with normal ovulation and normospermic partners. *Fertility and Sterility*.

[3] Canis M., Donnez J. G., Guzick D. S. (1997). Revised American Society for Reproductive Medicine classification of endometriosis: 1996. *Fertility and Sterility*.

[4] Taylor H. S., Adamson G. D., Diamond M. P. (2018). An evidence-based approach to assessing surgical versus clinical diagnosis of symptomatic endometriosis. *International Journal of Gynecology & Obstetrics*.

[5] Moore J., Copley S., Morris J., Lindsell D., Golding S., Kennedy S. (2002). A systematic review of the accuracy of ultrasound in the diagnosis of endometriosis. *Ultrasound in Obstetrics & Gynecology*.

[6] Kennedy S., Bergqvist A., Chapron C. (2005). ESHRE guideline for the diagnosis and treatment of endometriosis. *Human Reproduction*.

[7] Dunselman G. A. J., Vermeulen N., Becker C. (2014). ESHRE guideline: management of women with endometriosis. *Human Reproduction*.

[8] Simoens S., Dunselman G., Dirksen C. (2012). The burden of endometriosis: costs and quality of life of women with endometriosis and treated in referral centres. *Human Reproduction*.

[9] Fassbender A., Vodolazkaia A., Saunders P. (2013). Biomarkers of endometriosis. *Fertility and Sterility*.

[10] Seeber B., Sammel M. D., Fan X. (2008). Panel of markers can accurately predict endometriosis in a subset of patients. *Fertility and Sterility*.

[11] Vodolazkaia A., El-Aalamat Y., Popovic D. (2012). Evaluation of a panel of 28 biomarkers for the non-invasive diagnosis of endometriosis. *Human Reproduction*.

[12] Rižner T. L. (2014). Noninvasive biomarkers of endometriosis: myth or reality?. *Expert Review of Molecular Diagnostics*.

[13] May K. E., Conduit-Hulbert S. A., Villar J., Kirtley S., Kennedy S. H., Becker C. M. (2010). Peripheral biomarkers of endometriosis: a systematic review. *Human Reproduction Update*.

[14] May K. E., Villar J., Kirtley S., Kennedy S. H., Becker C. M. (2011). Endometrial alterations in endometriosis: a systematic review of putative biomarkers. *Human Reproduction Update*.

[15] Hall J., D'Hooghe T. (2017). Building translational research infrastructure and access to expertise for biomarker discovery in cancer. *Biomarkers for Endometriosis*.

[16] Rifai N., Gillette M. A., Carr S. A. (2006). Protein biomarker discovery and validation: the long and uncertain path to clinical utility. *Nature Biotechnology*.

[17] Rahmioglu N., Fassbender A., Vitonis A. (2014). World endometriosis research foundation endometriosis phenome and biobanking harmonization project: III. Fluid biospecimen collection, processing, and storage in endometriosis research. *Fertility and Sterility*.

[18] Fassbender A., Rahmioglu N., Vitonis A. (2014). World endometriosis research foundation endometriosis phenome and biobanking harmonisation project: IV. Tissue collection, processing, and storage in endometriosis research. *Fertility and Sterility*.

[19] Palmer S. S., Barnhart K. T. (2013). Biomarkers in reproductive medicine: the promise, and can it be fulfilled?. *Fertility and Sterility*.

[20] Aziz N., Nishanian P., Mitsuyasu R., Detels R., Fahey J. L. (1999). Variables that affect assays for plasma cytokines and soluble activation markers. *Clinical and Vaccine Immunology*.

[21] Vitonis A. F., Vincent K., Rahmioglu N. (2014). World endometriosis research foundation endometriosis phenome and biobanking harmonization project: II. Clinical and covariate phenotype data collection in endometriosis research. *Fertility and Sterility*.

[22] Becker C. M., Laufer M. R., Stratton P. (2014). World endometriosis research foundation endometriosis phenome and biobanking harmonisation project: I. Surgical phenotype data collection in endometriosis. *Fertility and Sterility*.

[23] Hinkle D. E., Wiersma W., Jurs S. G. (2003). *Applied Statistics for the Behavioral Sciences*.

[24] Claudon A., Vergnaud P., Valverde C., Mayr A., Klause U., Garnero P. (2008). New automated multiplex assay for bone turnover markers in osteoporosis. *Clinical Chemistry*.

[25] D'Hooghe T. M., Mihalyi A. M., Simsa P. (2006). Why we need a noninvasive diagnostic test for minimal to mild endometriosis with a high sensitivity. *Gynecologic and Obstetric Investigation*.

[26] Duffy M. J., Sturgeon C. M., Soletormos G. (2015). Validation of new cancer biomarkers: a position statement from the european group on tumor markers. *Clinical Chemistry*.

[27] Nisenblat V., Bossuyt P. M. M., Shaikh R. (2016). Blood biomarkers for the non-invasive diagnosis of endometriosis. *Cochrane Database of Systematic Reviews*.

[28] Moss E. L., Hollingworth J., Reynolds T. M. (2005). The role of CA125 in clinical practice. *Journal of Clinical Pathology*.

[29] Bast R. C., Skates S., Lokshin A., Moore R. G. (2012). Differential diagnosis of a pelvic mass: improved algorithms and novel biomarkers. *International Journal of Gynecological Cancer*.

[30] DF O., Flores I., Waelkens E., D'Hooghe T. (2018). Noninvasive diagnosis of endometriosis: Review of current peripheral blood and endometrial biomarkers. *Best Practice & Research Clinical Obstetrics & Gynaecology*.

[31] Taylor J. M., Ankerst D. P., Andridge R. R. (2008). Validation of biomarker-based risk prediction models. *Clinical Cancer Research*.

[32] Sperandei S. (2014). Understanding logistic regression analysis. *Biochemical Medicine*.

[33] Harrell F. E., Lee K. L., Mark D. B. (1996). Multivariable prognostic models: issues in developing models, evaluating assumptions and adequacy, and measuring and reducing errors. *Statistics in Medicine*.

