# Supplementary material for: Technical Verification and Assessment of Independent Validation of Biomarker Models for Endometriosis
Source: Biomed Res Int. 2019 Jul 25;2019:3673060. doi: 10.1155/2019/3673060 (PMC6683797; doi:10.1155/2019/3673060)
Supplement: Supplementary Materials — Supplementary Table I. Levels of plasma biomarkers for ultrasound-negative endometriosis versus controls. [file 3673060.f1.docx]

**Supplementary Table I.** **Levels of plasma biomarkers for ultrasound-negative endometriosis versus controls**

|  |  | Technical verification study | | | Validation study | | |
| --- | --- | --- | --- | --- | --- | --- | --- |
| Biomarker | Phase of cycle | Control | Endometriosis (US neg) | p-value | Control | Endometriosis (US neg) | p-value |
| CA-125 (U/ml) | All (no med) | 15.61  (11.38-22.52) | 17.75  (12.11-24.17) | NS | 13.00  (9.000- 16.00) | 20.00  (13.00-27.00) | **** |
|  | Menstrual | 19.49  (12.39-27.42) | 19.33  (14.35-31.76) | NS | 16.00  (13.00- 23.50) | 22.00  (13.00-41.00) | NS |
|  | Follicular | 11.97  (9.859-16.13) | 16.65  (11.94-24.10) | NS | 11.00  (8.000-17.00) | 20.00  (11.00-27.00) | * |
|  | Luteal | 16.99  (12.62-25.34) | 15.47  (11.40-23.38) | NS | 12.00  (9.500- 13.00) | 18.50  (13.50-23.75) | ** |
|  | Medication | N/A | N/A | N/A | 9.000  (5.000- 12.00) | 11.50  (7.750-16.50) | NS |
| VEGF (pg/ml) | All (no med) | 41.44  (27.49- 58.59) | 40.05  (28.39-65.65) | NS | 404.6  (296.5-545.1) | 426.8  (299.5-518.5) | NS |
|  | Menstrual | 43.69  (29.50- 54.13) | 43.27  (30.56-68.32) | NS | 414.3  (364.5-507.0) | 481.7  (371.5-550.4) | NS |
|  | Follicular | 34.90  (25.17- 47.24) | 40.27  (25.56-65.65) | NS | 400.0  (278.5-581.5) | 415.3  (252.6-516.2) | NS |
|  | Luteal | 56.22  (22.36-98.14) | 36.71  (25.70-64.76) | NS | 399.3  (312.3-568.1) | 414.4  (320.2-483.7) | NS |
|  | Medication | N/A | N/A | N/A | 368.7  (217.2-490.4) | 355.6  (268.7-423.5) | NS |
| Annexin V (ng/ml) | All (no med) | 15.51  (11.41-20.56) | 12.71  (9.070-20.36) | NS | 7.322  (3.568-49.06) | 12.46  (3.822-51.16) | NS |
|  | Menstrual | 15.69  (13.94-18.17) | 11.08  (9.520-22.31) | NS | 4.519  (2.031-25.69) | 11.20  (3.988-36.55) | NS |
|  | Follicular | 15.06  (11.16-18.06) | 12.71  (6.520-20.02) | NS | 9.320  (3.097-24.11) | 12.78  (3.769-56.31) | NS |
|  | Luteal | 17.13  (8.490-24.31) | 14.36  (9.505-19.42) | NS | 17.05  (3.881-63.47) | 12.46  (3.364-60.64) | NS |
|  | Medication | N/A | N/A | N/A | 17.41  (4.489-57.69) | 7.076  (2.910-14.23) | NS |
| sICAM-1 (ng/ml) | All (no med) | 154.8  (137.4-182.1) | 150.9  (131.7- 195.7) | NS | 209.5  (183.3- 236.2) | 201.0  (169.8- 219.5) | NS |
|  | Menstrual | 176.0  (135.0-246.9) | 172.4  (133.2- 297.3) | NS | 195.7  (186.0-230.2) | 198.2  (157.1- 208.1) | NS |
|  | Follicular | 149.0  (130.7-170.8) | 150.9  (129.2- 208.8) | NS | 207.9  (171.0-228.9) | 208.3  (168.3- 231.0) | NS |
|  | Luteal | 155.7  (134.1-177.1) | 144.5  (131.7- 166.1) | NS | 227.4  (189.1-241.2) | 191.0  (173.3- 216.9) | NS |
|  | Medication | N/A | N/A | N/A | 227.4  (197.7-260.3) | 187.0  (161.3- 212.3) | * |
| Glycodelin (ng/ml) | All (no med) | 29.06  (11.90-47.50) | 35.11  (14.12- 89.01) | NS | 3.237  (1.254-11.30) | 6.253  (2.746- 21.66) | ** |
|  | Menstrual | 45.68  (37.62-109.1) | 110.4  (56.59- 190.7) | NS | 7.242  (2.312-19.86) | 24.68  (12.25- 40.01) | 0.05 |
|  | Follicular | 12.87  (9.850-23.80) | 16.41  (10.08- 34.98) | NS | 1.573  (0.9660-7.578) | 5.717  (2.581- 11.89) | NS |
|  | Luteal | 31.13  (11.91-54.83) | 42.99  (17.60- 118.7) | NS | 5.026  (1.513-11.30) | 3.964  (2.109- 21.84) | NS |
|  | Medication | N/A | N/A | N/A | 0.9502  (0.6382-3.436) | 1.580  (0.8736- 2.950) | NS |

Data are presented as the median and interquartile range. Mann-Whitney test was performed for all phases combined, while Kruskal-Wallis with post-hoc Dunn’s analysis was done when biomarkers were analyzed according to cycle phase. No med = no medication. NS = not significant. N/A indicates that there was no medication cohort included in the technical verification study
